# Gypenoside LXXV Promotes Cutaneous Wound Healing In Vivo by Enhancing Connective Tissue Growth Factor Levels Via the Glucocorticoid Receptor Pathway

**DOI:** 10.3390/molecules24081595

**Published:** 2019-04-23

**Authors:** Sungjoo Park, Eunsu Ko, Jun Hyoung Lee, Yoseb Song, Chang-Hao Cui, Jingang Hou, Byeong Min Jeon, Hun Sik Kim, Sun Chang Kim

**Affiliations:** 1 Department of Biological Sciences, Korea Advanced Institute of Science and Technology, Daejeon 34141, Korea; parksungjoo@kaist.ac.kr (S.P.); eunsu_93@kaist.ac.kr (E.K.); junhlee@kaist.ac.kr (J.H.L.); jbm0901@kaist.ac.kr (B.M.J.); 2 Department of Biological Sciences and KI for the BioCentury, Korea Advanced Institute of Science and Technology, Daejeon 34141, Korea; yosebiback@kaist.ac.kr; 3 Intelligent Synthetic Biology Center, Daejeon 34141, Korea; seldoms@163.com (C.-H.C.); houjingang1225@126.com (J.H.); 4 Department of Biomedical Sciences, University of Ulsan College of Medicine, Asan Institute for Life Sciences, Asan Medical Center, Seoul 05505, Korea; hunkim@amc.seoul.kr

**Keywords:** ginsenoside, gypenoside LXXV, wound healing, connective tissue growth factor, glucocorticoid receptor, RNA sequencing

## Abstract

Cutaneous wound healing is a well-orchestrated event in which many types of cells and growth factors are involved in restoring the barrier function of skin. In order to identify whether ginsenosides, the main active components of *Panax ginseng*, promote wound healing, the proliferation and migration activities of 15 different ginsenosides were tested by MTT assay and scratched wound closure assay. Among ginsenosides, gypenoside LXXV (G75) showed the most potent wound healing effects. Thus, this study aimed to investigate the effects of G75 on wound healing in vivo and characterize associated molecular changes. G75 significantly increased proliferation and migration of keratinocytes and fibroblasts, and promoted wound closure in an excision wound mouse model compared with madecassoside (MA), which has been used to treat wounds. Additionally, RNA sequencing data revealed G75-mediated significant upregulation of connective tissue growth factor (CTGF), which is known to be produced via the glucocorticoid receptor (GR) pathway. Consistently, the increase in production of CTGF was confirmed by western blot and ELISA. In addition, GR-competitive binding assay and GR translocation assay results demonstrated that G75 can be bound to GR and translocated into the nucleus. These results demonstrated that G75 is a newly identified effective component in wound healing.

## 1. Introduction

The skin is the largest organ and mainly functions as a barrier between the outer and inner environments [1]. Cutaneous wound healing is an important process in that a loss of the skin’s barrier function following wounds is restored by interplay and crosstalk between various cells and mediators [2]. Wound healing occurs in three stages: inflammation, proliferation, and remodeling [3]. The proliferation stage occurs sequentially to restore the epithelial barrier function by stimulating the proliferation and migration of cells, such as keratinocytes and fibroblasts [4]. The proliferation and migration of both these cells are the most critical processes in wound healing because re-epithelization accounts for up to 80% of wound closure [2,5]. Failure to regain the barrier function may induce wound reoccurrence, which further complicates the clinical situation [6]. Since cutaneous wound healing is important not only for clinical problems, but also for aesthetic reasons, the effects of various bioactive components on promoting wound healing have been studied [7,8,9].

*Panax ginseng* C.A. Meyer is considered as one of the highly valued herbs for enhancing health, and it has long been traditionally used [10]. Ginsenosides are the major pharmacologically active compounds in *Panax ginseng* [11]; and showed various pharmaceutical activity including wound healing [12,13,14]. For example, ginsenoside Rb1 promotes wound healing via the p38MAPK/MSK2/NF-κB pathway [9] and exhibits beneficial burn wound-healing action [15]. In addition, ginsenoside Rd significantly decreased the size of excision wounds in vivo and accelerated wound healing process in laser burn wounds [16]. Although studies have reported the wound healing effects of ginsenoside, few studies have been performed on such effects mediated by their derivatives. Furthermore, the molecular mechanism underlying wound healing by ginsenosides remains unclear.

Gypenoside LXXV (G75), a protopanaxadiol type, is formed by deglycosylation from Rb1. Due to its low content in ginseng, the pharmacological effects of G75 remain largely unknown [17]. In this study, the wound healing activities of 15 different ginsenosides were tested in vitro. Furthermore, we investigated the wound healing effects of G75 in vitro and in vivo compared with MA and examined the potential mechanism of G75 action in wound healing.

## 2. Results

### 2.1. Effect of Different Types of Ginsenosides on the Proliferation and Migration of HaCaT Keratinocytes

**Figure 1:**
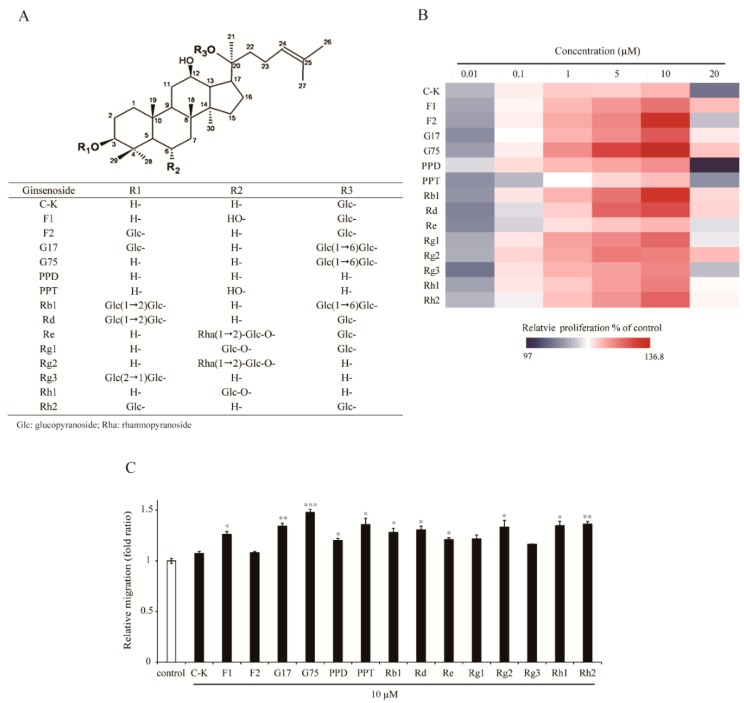
Chemical structure of (**A**) ginsenoides. (**B**,**C**) Comparison of the effect of various ginsenosides on HaCaT keratinocyte cell proliferation and migration. (**B**) HaCaT cells were treated with various concentrations of ginsenosides (0.01–20 μM) or dimethyl sulfoxide (DMSO, vehicle control) for 48 h. Cell proliferation was then evaluated using MTT assay. Heatmaps showed the relative proliferation induced by different types of ginsenosides. Colors indicate relative proliferation values from the lowest (blue) to the highest (red). (**C**) Scratch wounds were created on monolayer of HaCaT cells, and photographed immediately after wounding (0 h) using phase-contrast microscopy. Cells were incubated with 10 μM of each indicated ginsenoside for 24 h and photographed (24 h). Relative migration was calculated by analyzing the measured wound closure area. DMSO was used as a vehicle control. The bars represent the mean ± SEM of three independent assays. * *p* < 0.05, ** *p* < 0.01, *** *p* < 0.001, two-tailed Student’s *t*-test.

Proliferation and migration of keratinocytes are important processes that induce efficient wound closure. To test the capacity of ginsenosides to induce proliferation and migration of HaCaT keratinocytes, cells were exposed to 15 different ginsenosides (Figure 1A) (C-K, F1, F2, gypenoside XVII (G17), G75, protopanaxadiol (PPD), protopanaxatriol (PPT), Rb1, Rd, Re, Rg1, Rg2, Rg3, Rh1 and Rh2) and cell proliferation was evaluated by MTT assay. As shown in Figure 1B, diverse ginsenosides were capable of inducing cell proliferation in a dose-dependent manner at a concentration range from 0.01 µM to 10 µM (F1, from 5.5% to 24.4%; F2, from 5.2% to 34.7%; G17, from 4.0% to 30.0%; G75, from 5.1% to 36.8%; PPD, from 8.3% to 20.5%; PPT, from 4.0% to 15.3%; Rb1, from 4.5% to 33.3%; Rd, from 3.3% to 29.3%; Rg1, from 5.8% to 26.0%; Rg3, from 2.3% to 21.7%; Rh1, from 5.4% to 22.8%; Rh2, from 6.4% to 26.6% increased, respectively). Cell proliferation was the highest at 10 µM, but slightly decreased at 20 µM of various ginsenosides. Among other ginsenosides, G75 showed the most prominent effects on proliferation of HaCaT keratinocytes.

To determine cell migration efficiency of 15 kinds of ginsenosides, a scratch wound closure assay was performed (Figure 1C). Migration of cells into the wounded area was significantly increased in the presence of ginsenosides (10 µM) compared with control within 24 h after wounding. Of tested ginsenosides, F1, G17, G75, PPD, PPT, Rg2, Rh1, Rh2, Rb1, Rd, and Re significantly induced migration of HaCaT keratinocytes. G75 showed the strongest effects on migration of HaCaT keratinocytes. These results suggested that G75 has most promising wound healing activity among different kinds of ginsenosides in terms of proliferation and migration of HaCaT keratinocytes.

### 2.2. Effect of G75 on Wound Healing In Vitro

**Figure 2:**
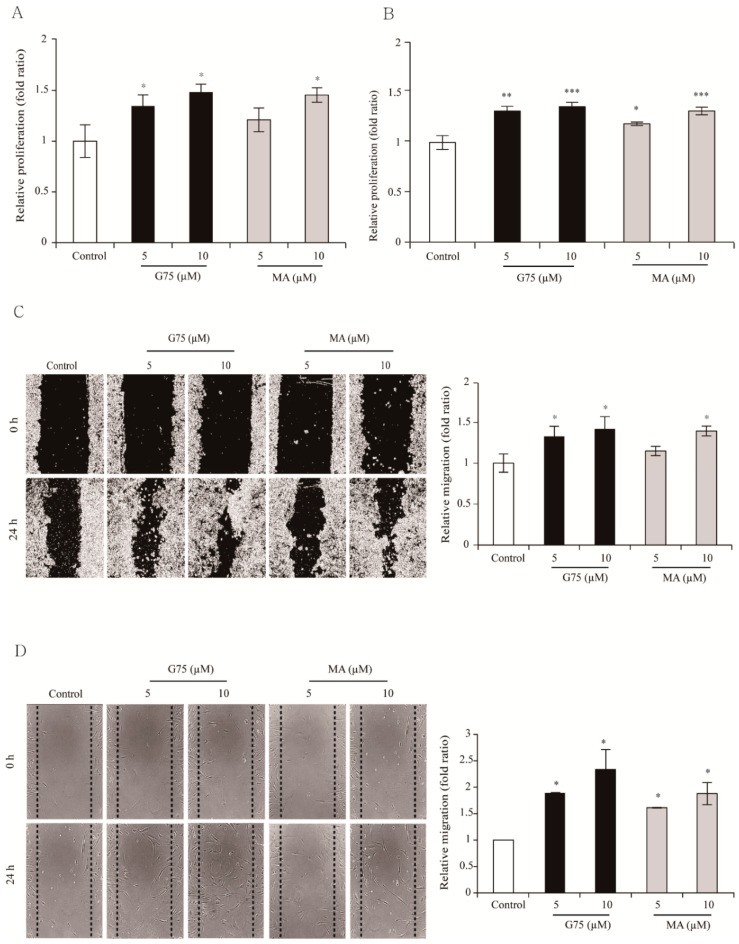
Wound healing effects of G75 in vitro. G75 induced cell proliferation in (**A**) HaCaT and (**B**) fibroblasts. After incubation for 48 h with indicated concentrations of G75 or MA, cell viability was assessed using MTT assay. G75 induced migration in (**C**) HaCaT and (**D**) fibroblasts. In vitro wound closure was assessed by a scratch wound closure assay. Wound closure after 24 h of incubation was calculated. The results are presented as means ± S.E.M (*n* = 3 wells per condition). * *p* < 0.05, ** *p* < 0.01, *** *p* < 0.001, two-tailed Student’s *t*-test.

To further evaluate the potential wound healing activity of G75, we first compared G75 with MA, which is known to have significant wound healing activities at a concentration of 10 µM [18], in terms of in vitro proliferation of HaCaT and fibroblasts. MA has potential wound healing activities, so it is one of main reasons for the use of *Centella asiatica* herbs to treat wounds [19]. As shown in Figure 2A,B, G75 induced significant proliferation at a tested concentration of 5 µM and 10 µM in both cell lines, resulting in insignificant difference between the two concentrations. In addition, the migration assay showed that G75 could induce cell migration of HaCaT keratinocytes and fibroblasts at 5 µM and 10 µM (Figure 2C,D). In comparison, MA increased proliferation and migration significantly only at 10 µM concentration (Figure 2A,D), suggesting G75 is more potent than MA for wound healing in vitro.

### 2.3. Effect of G75 on Wound Healing In Vivo

**Figure 3:**
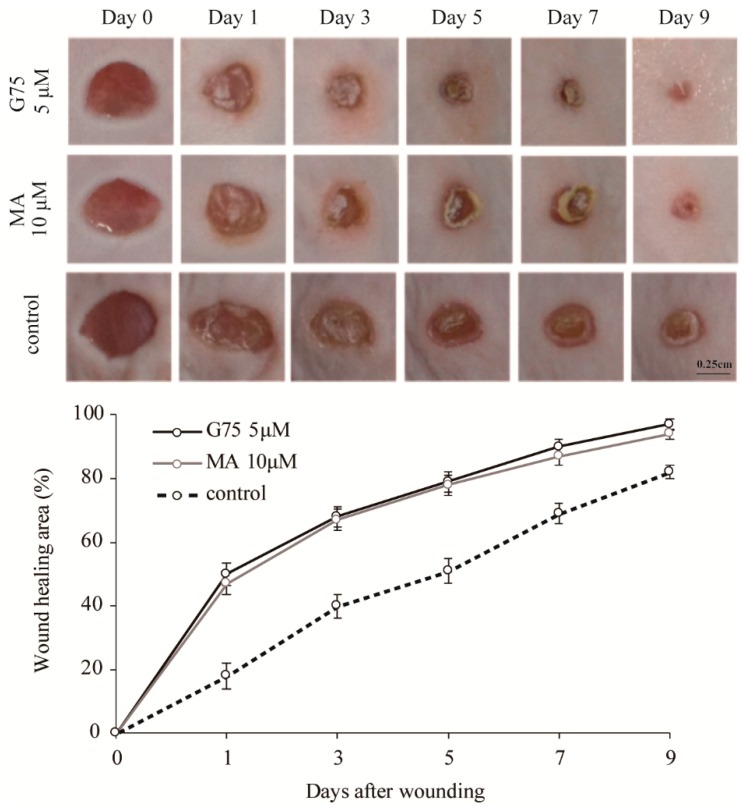
Effects of G75 on excision wound healing in mice. After the excision wound was made using a 5-mm diameter biopsy punch, either 5 µM of G75 or 10 µM of MA was applied to the wound surface. DMSO was used as vehicle control. The wound healing area was measured on indicating days from the day of wounding. The results are presented as means ± S.E.M (*n* = 6).

To verify the effects of G75 on wound healing, in vivo wound closure activity of G75 in an excision wound mouse model was evaluated. Two full-thickness round wounds of 5 mm in diameter were made on the dorsal back skin of mice, and the changes of wound closure were evaluated by measurement of original wound area (%). G75 (5 µM/wound) or MA (10 µM/wound) was applied topically once a day for 9 days post-injury. DMSO was used as a vehicle control for the G75 and MA treatments. In consistent with in vitro results, wounds after treatment with G75 and MA were smaller than wounds treated with vehicle control (Figure 3). The enhancement of wound healing became apparent from a day after the initiation of treatment and most evident after three days. Treatment with 5 µM of G75 exhibited a statistically significant effect on wound closure on days 1, 3, 5, 7, and 9 after treatments, respectively. Insignificant difference between G75 and MA-applied wound areas in mice was identified (Figure 3). Based on the observation, G75 has similar or more potent effect than MA on skin wound closure.

### 2.4. Effect of G75 on Biological Processes in Wound Healing

The molecular mechanism of wound healing process by ginsenosides is still largely unknown. Therefore, to investigate the molecular mechanism of G75 associated with wound healing, RNA sequencing was performed using next generation sequencing (NGS). For sequencing, RNA isolated from the dorsal back excisional wound tissue on the third day after each treatment was sequenced. A significant change was defined as a 1.7-fold change and when the difference had a *p*-value lower than 0.05. We obtained 1048 significantly upregulated genes by G75 treatment compared to vehicle control.

**Figure 4:**
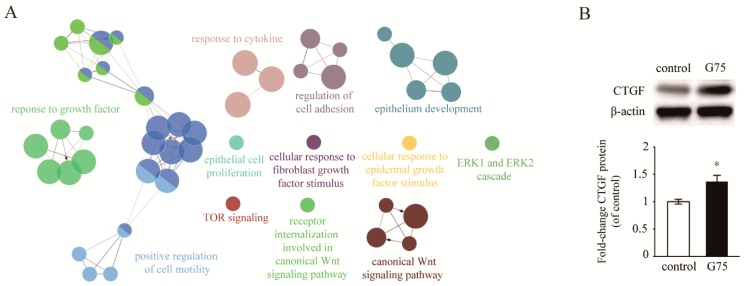
Effects of G75 on the biological pathways involved in wound healing. (**A**) Significantly activated biological processes on day three of G75 treatment based on the enriched GO clusters visualized by ClueGO are shown. To reduce redundancy of GO terms, fusion option was selected. For the functionally grouped networks with terms as nodes, the size of nodes represents the term enrichment significance linked with edge size based on their kappa score level (≥0.4), in which only the label of the most significant term per group is shown. (**B**) CTGF expression was increased by G75. Representative western blot analysis of CTGF expression in skin tissues collected from G75-treated mice three days after wounding. The results are presented as means ± S.E.M (*n* = 3). * *p* < 0.05, two-tailed Student’s *t*-test.

To identify significantly changed biological functions, enriched gene ontology (GO) biological processes were analyzed by comparing the up-regulated genes mediated by G75 using ClueGO [20]. We found a clear enrichment of genes involved in wound healing such as a response to growth factor, cell migration, positive regulation of cell motility, response to cytokine, regulation of cell adhesion, epithelium development, epithelial cell proliferation, cellular response to fibroblast growth factor stimulus, cellular response to epidermal growth factor stimulus, ERK1 and ERK2 cascade, TOR signaling, and receptor internalization involved in the canonical Wnt signaling pathway (Figure 4A). These results indicated that G75 showed wound healing activity by inducing several kinds of signaling pathways related to growth factors.

To identify the significant growth factor affecting wound healing by G75, the mRNA level of growth factors was analyzed based on RNA sequencing data (Table 1). In particular, CTGF was significantly up-regulated by G75 among all these genes. To further confirm the upregulation of CTGF after G75 treatment, western blot analysis was performed. Lysates were isolated from the dorsal back excision wound tissue on the third day after G75 treatment. As shown in Figure 4B, CTGF was significantly upregulated in G75-treated wound tissue compared with vehicle control.

### 2.5. G75 Induces CTGF Production Via GR Pathway in Fibroblasts

**Figure 5:**
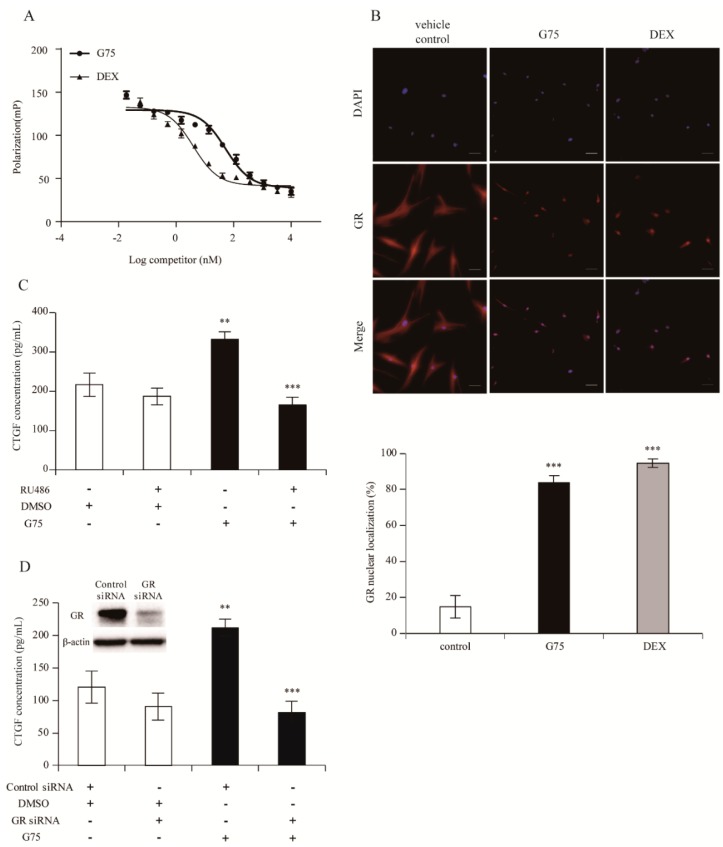
G75-mediated CTGF production through the GR pathway in fibroblasts. (**A**) G75 competitively bound to GR. DEX was used as positive control. Fluorescent polarization is expressed in millipolarization (mP). Data represent the mean from three independent experiments. (**B**) G75 induced GR translocation into the nucleus. Fibroblasts were incubated with G75 (5 µM) or DEX (1 µM) for 24 h and immunostained with GR antibody (red) and nucleus was stained with DAPI (blue). Original magnification was 400×. Relative GR translocation is assessed as described in experimental method section. (**C**) Fibroblasts pretreated with RU486 (10 µM) for 30 min were incubated with G75 (5 µM) for another 24 h. (**D**) Fibroblasts pre-treated with GR siRNA (10 nM) or control siRNA (10 nM) for 48 h were incubated with DMSO, or G75 (5 µM) for another 24 h. All experiments were carried out in triplicate. The results are presented as means ± S.E.M (*n* = 3). ** *p* < 0.01, *** *p* < 0.001, two-tailed Student’s *t*-test.

Since CTGF is known to be induced by the GR pathway [21], to elucidate whether G75 produces CTGF via the GR pathway by direct interaction with GR and translocation, GR-competitive ligand-binding assay and GR-translocation assay were performed. The results indicated that G75 is capable of binding to GR. The IC_50_ value for G75 and dexamethasone (DEX, positive control) were 52.84 nM and 4.00 nM, respectively (Figure 5A). In addition, we attempted to explore the efficacy of G75 to induce GR nuclear translocation in fibroblasts. As shown in Figure 5B, GR was translocated by G75 and DEX (83.6% and 94.6%, respectively). These results showed the possibility that G75 binds to GR and induces GR translocation into nucleus at the cellular level.

To further understand whether G75 induces CTGF via the GR pathway, we quantified the production of CTGF via ELISA assay in the presence of RU486 or GR siRNA. Successful knockdown of GR was conducted using GR siRNA and confirmed by western blot (Figure 5D). As shown in Figure 5C,D, ELISA assay revealed that CTGF production was significantly elevated by G75 treatment in the culture supernatant of human dermal fibroblasts, by contrast, G75-mediated increase of CTGF level was significantly reduced by treatment with RU486 or GR siRNA (Figure 5C,D). These results demonstrated that G75 induces CTGF via the GR-dependent pathway.

## 3. Discussion

As previously mentioned in the literature, ginsenosides have various pharmacological activities including wound healing [9,15,16,22,23,24,25]. On the basis of these previous studies, we hypothesized that G75, a deglycosylated form of ginsenoside Rb1, has beneficial effects on wound healing. It showed a notable efficacy in inducing proliferation and migration of keratinocytes among 15 types of ginsenosides. Furthermore, G75 significantly induced proliferation and migration of keratinocytes and fibroblasts at a lower concentration (5 μM) than MA in vitro and in vivo. These results indicated that G75 can be a useful candidate compound in cutaneous wound healing.

Diverse signaling pathways and molecular mediators play a key role in wound healing [26,27]. To understand the molecular mechanism of wound healing by G75, we analyzed gene expression associated with wounds treated with G75, and the analysis showed that topical application of G75 accelerated excision wound healing by positively influencing various events related to wound healing. All these identified signaling pathways activated by G75 have been known to be regulated by growth factors during the wound healing process [28,29,30,31,32,33].

Many studies have reported that growth factors play key roles in tissue repair wherein growth factors stimulate cell proliferation and migration into the wound area, resulting in clinically promoting wound healing [34,35]. Therefore, we focused on the effects of G75 on the expression of growth factors. Among several growth factors, CTGF, which is involved in the CCN family and called CCN2 [33], was the only one that was induced significantly by G75. CTGF plays a role in various biological actions, such as cell migration, proliferation, angiogenesis, and early stage of wound healing [36,37]. According to previous reports, CTGF is regulated by GR [21] and ginsenosides have the potential to act as ligands of GR [38,39]. Thus, we hypothesized that, in the process of wound healing, G75 triggers CTGF production after binding to GR and translocating into the nucleus. Our results showed that G75 possibly binds to GR and induces its translocation from cytoplasm to nucleus. In addition, G75 treatment increased CTGF production; meanwhile, RU486 treatment and GR knockdown induced interference of CTGF production. These results suggested that G75 contributes to wound healing by producing CTGF at least in part via the GR-dependent pathway.

In conclusion, we explored the wound healing properties of G75 and its potential mechanism of action. Although the exact mechanism underlying wound healing activity of G75 remains to be further examined, our results suggest that G75 has potent wound healing activities, and a different mechanism compared with MA and Rb1 in wound healing [18,40]. Taken together, G75 is a new potential treatment for skin wound healing and is expected to have a synergistic effect with other compounds.

## 4. Materials and Methods

### 4.1. Cell Culture and Reagents

HaCaT (human keratinocytes) (Cell Line Service, Eppelheim, Germany) and human dermal fibroblasts (CCD986sk, ATCC, Manassas, VA, USA) were incubated in DMEM (Thermo Fisher Scientific, Waltham, MA, USA), supplemented with 10% heat-inactivated FBS, 100 U/mL penicillin, and 100 μg/mL streptomycin, at 37 °C and humidified 5% CO_2_ atmosphere.

Ginsenoside C-K, F1, F2, G17, G75, PPD, PPT, Rg2, Rg3, Rh1, and Rh2 (>95% pure) were prepared using enzymatic methods as previously reported [17,41,42,43,44,45,46,47]. Ginsenosides Rb1, Rd, Re, and Rg1 were directly purified from protopanaxadiol (Hongjiu Biotech Co., Ltd., Dalian, China) type or protopanaxatriol (Da Nature Biological Engineering Co., Ltd., Fusong, China) type ginsenoside mixtures using a silica column (168 × 71 mm id, Biotage, Uppsala, Sweden), an ODS column (157 × 39 mm id, Biotage, Uppsala, Sweden), and recycling preparative HPLC (>95% purity). Ginsenosides were dissolved in dimethyl sulfoxide (DMSO). Madecassoside (MA) was purchased from Changzhou United Chemical Co., Ltd. (Changzhou, China). RU486 (GR antagonist) and DEX (GR agonist) were purchased from Sigma-Aldrich Co. (St. Louis, MO, USA). Control siRNA and GR siRNA were purchased from Cell Signaling Technology (Danvers, MA, USA).

### 4.2. Cell Proliferation Assay

Cell proliferation was determined using CellTiter 96 Non-radioactive Cell proliferation assay kit (Promega, Madison, WI, USA) according to manufacturer’s instructions. Briefly, cells were seeded in 96-well culture plates and incubated for 48 h at 37 °C under a humidified 5% CO_2_ atmosphere. Then, the cells were treated with 5 or 10 μM of G75 and MA and DMSO (vehicle control) diluted in DMEM without FBS for 48 h. Absorbance of formazan crystals was read at 490 nm using a microplate reader (Spark, Tecan, Switzerland) after adding MTT (3-(4,5-dimethylthiazol-2-yl)-2,5-diphenyl tetrazolium bromide) solution.

### 4.3. Scratch Wound Closure Assay

HaCaT and fibroblasts were cultured in a 6-well culture plate for 24 h until 90% confluence was obtained. Cells were scraped off from the bottom of a 6-well culture plate using a 200 μL micropipette tip to create a cell-free area. After removing the cell debris with PBS washing, cells were incubated with 5 or 10 μM of G75 and MA and DMSO (vehicle control) diluted in DMEM without FBS for 48 h. Images were photographed under a microscope at 40× magnification. The gap at 0 h (immediately after scratch wound) was considered as 100%, and cellular migration was determined and compared with that after 24 h. The average area of the gaps was calculated using the Image J program (NIH, Bethesda, ME, USA).

### 4.4. Animals and Excisional Wound Mouse Model

Eight-week-old male ICR mice were purchased from Narabiotech (Pyeongtaek, Korea) and acclimatized for one week. The animal house was maintained at 22 ± 3 °C, 50 ± 10% humidity, and a 12-h light-dark cycle. All animals were freely allowed equal access to food and water. All animal procedures were performed according to the guidelines of the Institutional Animal Care and Use Committee (IACUC) at Korea Advanced Institute of Science and Technology (Daejeon, Korea).

The mice were randomized into various groups (6 mice per group). The excisional wound mouse model was established according to a previous method [48] with minor modifications. Briefly, dorsal hair was shaved by using a hair clipper and depilatory cream. Dorsal skin was disinfected with 70% ethanol. Then, the animal was placed in a lateral position and a 5-mm diameter circular wound was created using a sterile biopsy punch to remove two layers of dorsal skin and create full-thickness excisional wounds. Test materials (5 μM G75 (>95% purity, Figure S1), 10 μM MA, or DMSO) were dissolved in propylene glycol and then applied on the wound area once daily for nine days. The wound areas were calculated on days 0, 1, 3, 5, 7, and 9 using the Image J program (NIH, Bethesda, ME, USA), where the perimeter was established, and the contraction rate was calculated by the formula:

Rate of contraction (%) = (Area on day 0 − Area on day evaluated)/(Area on day 0)

### 4.5. NGS Analysis

Skin tissues obtained from the excisional wound mouse model on day 3 after wounding were ground and homogenized completely using a pestle with liquid nitrogen. Total RNA was isolated using RNeasy mini spin column (Qiagen, Hilden, Germany), following the manufacturer’s instructions. All RNA sequencing and alignment procedures were conducted by ChunLab (Seoul, Korea). The obtained RNA was used to construct an Illumina platform sequencing library, according to the manufacturer’s instructions. The constructed libraries were sequenced using paired-end 100-bp size Illumina HiSeq 2500 kit. Following the sequencing, the generated reads were trimmed based on high quality, then the trimmed reads were mapped onto the reference genome retrieved from the NCBI database using Bowtie2. The mapped reads were normalized to identify the relative transcript abundance based on fragments in reads per kilobase of exon sequence per million mapped sequence reads (FPKM). Visualization of mapping results and differentially expressed gene (DEG) analysis were performed using the CLRNASeq^TM^ program (ChunLab, Seoul, Korea). The differentially expressed genes were determined by a fold-change of greater or less than 1.7 and a *p*-value less than 0.05. The significantly upregulated genes were used for ClueGO analysis [20].

### 4.6. Western Blot Analysis

Skin tissue samples of mice were obtained and ground into homogenates. The homogenates were collected in microcentrifuge tubes, and lysed with phenylmethylsulfonyl fluoride (RIPA) and phosphatase inhibitor; 100:1) for 20 min. Then the homogenates were sonicated and centrifuged. The protein concentration of the tissue was determined using a protein measurement solution (PRO-MEASURE; iNtRON, Korea) and BSA as a standard. Total protein extracts were electrophoretically separated using SDS-PAGE (12% gel) and transferred onto polyvinylidene difluoride membranes (Invitrogen, Carlsbad, CA, USA). After blocking with 5% skim milk, the membranes were incubated with the antibody against CTGF (1:1000 dilution; Santa Cruz Biotechnology, Dallas, TX, USA) for 2 h and additionally incubated with secondary peroxidase-linked anti-mouse or anti-rabbit IgG (1:1000 dilution; Cell Signaling Technology, Danvers, MA, USA). Protein bands were visualized by ECL solution (Pierce Biotechnology, Waltham, MA, USA) and band intensities were quantified via the Image J program (NIH, Bethesda, ME, USA).

### 4.7. ELISA

Fibroblasts were cultured on 96-well plates. Cells were pre-treated with RU486 (10 µM) for 30 min and then treated with 5 μM G75 for 24 h. For siRNA knockdown, cells were transfected with control siRNA, or GR siRNA (10 nM) for 48 h using Hiperfect (Qiagen, Hilden, Germany) according to the manufacturer’s instructions, then treated with G75 for 24 h. For the detection of CTGF, the supernatants were collected and the concentration of CTGF was determined by a Human CTGF ELISA kit (Peprotech, Rocky Hill, NJ, USA) according to the manufacturer’s instructions.

### 4.8. GR Binding Affinity Assay

The PolarScreen™ GR competitor assay (Thermo Fisher Scientific, Waltham, MA, USA) was used as per manufacturer’s instructions to determine the half-maximal competitive binding affinity, IC_50_. Fluorescence-labelled ligand (Fluormone) for GR and G75 were added to each reaction well of the plate and incubated for 2 h in the dark. Polarization values were measured via microplate reader (Spark, Tecan, Switzerland) with Ex 485 nm/Em 530 nm and plotted against the concentration of G75. The binding affinity of the standard ligand, DEX, was also measured. IC_50_ was determined by using a prism graph pad program (Ver.7.0, GraphPad Software, San Diego, CA, USA).

### 4.9. GR Translocation Assay

Fibroblasts were cultured on μ-dish 35 mm (ibidi GmbH, Planegg, Germany) for 24 h, then stimulated with DMSO (vehicle control), 5 μM G75, or 1 μM DEX for 24 h. Cells were fixed, permeabilized, and blocked using Immunofluorescence application solution kit (Cell Signaling Technology, Danvers, MA, USA) according to manufacturer’s instructions. Cells were treated with primary antibody GR (Cell Signaling Technology, Danvers, MA, USA) overnight at 4 °C and with secondary antibody (Alex Fluor 594, Cell Signaling Technology, Danvers, MA, USA) for 1 h at 22–25 °C. Cells were further stained with DAPI solution (Thermo Fisher Scientific, Waltham, MA, USA) for 5 min. Fluorescence microscopy was performed using a Nikon i2 U microscope (Tokyo, Japan). All images were processed in Nikon NIS-elements software (Ver. 4.0, Nikon, Tokyo, Japan). GR nuclear translocation was quantified as previously described by using Image J (NIH, Bethesda, ME, USA) [49]. Briefly, the percentage of the total corrected fluorescence of GR nuclear section per the total corrected fluorescence of total GR cellular section was calculated.

### 4.10. Statistical Analysis

Statistical comparisons between groups were made using unpaired two-sided *t*-tests, and differences with *p* < 0.05 (*), *p* < 0.005 (**), and *p* < 0.001 (***) were considered statistically significant.

## Figures and Tables

**Table 1:** List of G75-altered growth factor genes in excisional mouse model.

Growth Factors	Fold Change	*p*-Value
Ctgf (Connective tissue growth factor)	1.879	0.022 *
Angpt1 (Angiopoietin 1)	1.796	0.250
Fgf7 (Fibroblast growth factor 7)	1.789	0.070
Fgf10 (Fibroblast growth factor 10)	1.750	0.192
Tgf alpha (Transforming growth factor alpha)	1.504	0.278
Tgf beta1 (Transforming growth factor beta 1)	1.392	0.199
Igf1 (Insulin-like growth factor 1)	1.367	0.974
Vegf alpha (Vascular endothelial growth factor alpha)	1.281	0.343
Hbegf (Heparin binding EFG like growth factor)	1.267	0.685
Hgf (Hepatocyte growth factor)	1.191	0.826

* *p* < 0.05 compared with vehicle control.

## Supplementary Materials

The following are available online: Figure S1: HPLC results of the purified G75.

## References

[1] Sabol F., Dancakova L., Gal P., Vasilenko T., Novotny M., Smetana K., Lenhardt L. (2012). Immunohistological changes in skin wounds during the early periods of healing in a rat model. Vet. Med..

[2] Sorg H., Tilkorn D.J., Hager S., Hauser J., Mirastschijski U. (2017). Skin Wound Healing: An Update on the Current Knowledge and Concepts. Eur. Surg. Res..

[3] Grazul-Bilska A.T., Johnson M.L., Bilski J.J., Redmer D.A., Reynolds L.P., Abdullah A., Abdullah K.M. (2003). Wound healing: The role of growth factors. Drugs Today.

[4] Gonzalez A.C., Costa T.F., Andrade Z.A., Medrado A.R. (2016). Wound healing—A literature review. An. Bras. Dermatol..

[5] Qing C. (2017). The molecular biology in wound healing & non-healing wound. Chin. J. Traumatol..

[6] Pastar I., Stojadinovic O., Yin N.C., Ramirez H., Nusbaum A.G., Sawaya A., Patel S.B., Khalid L., Isseroff R.R., Tomic-Canic M. (2014). Epithelialization in Wound Healing: A Comprehensive Review. Adv. Wound Care.

[7] Kim S.K., Mendis E. (2006). Bioactive compounds from marine processing byproducts-A review. Food Res. Int..

[8] Lee J.H., Kim H.L., Lee M.H., You K.E., Kwon B.J., Seo H.J., Park J.C. (2012). Asiaticoside enhances normal human skin cell migration, attachment and growth in vitro wound healing model. Phytomedicine.

[9] Hou J., Kim S. (2018). Possible role of ginsenoside Rb1 in skin wound healing via regulating senescent skin dermal fibroblast. Biochem. Biophys. Res. Commun..

[10] Baeg I.H., So S.H. (2013). The world ginseng market and the ginseng. J. Ginseng Res..

[11] Lee D.Y., Lee Y.H., Yang D.C. (2010). Physicochemical Characterization and NMR Assignments of Ginsenosides Rb1, Rb2, Rc, and Rd Isolated from Panax ginseng. J. Ginseng Res..

[12] Sun M., Ye Y., Xiao L., Duan X., Zhang Y., Zhang H. (2017). Anticancer effects of ginsenoside Rg3. Int. J. Mol. Med..

[13] Nie L., Xia J., Li H., Zhang Z., Yang Y., Huang X., He Z., Liu J., Yang X. (2017). Ginsenoside Rg1 Ameliorates Behavioral Abnormalities and Modulates the Hippocampal Proteomic Change in Triple Transgenic Mice of Alzheimer’s Disease. Oxid. Med. Cell. Longev..

[14] Lee I.S., Uh I., Kim K.S., Kim K.H., Park J., Kim Y., Jung J.H., Jung H.J., Jang H.J. (2016). Anti-Inflammatory Effects of Ginsenoside Rg3 via NF-κB Pathway in A549 Cells and Human Asthmatic Lung Tissue. J. Immunol. Res..

[15] Kimura Y., Sumiyoshi M., Kawahira K., Sakanaka M. (2006). Effects of ginseng saponins isolated from Red Ginseng roots on burn wound healing in mice. Br. J. Pharmacol..

[16] Kim W.K., Song S.Y., Oh W.K., Kaewsuwan S., Tran T.L., Kim W.S., Sung J.H. (2013). Wound-healing effect of ginsenoside Rd from leaves of Panax ginseng via cyclic AMP-dependent protein kinase pathway. Eur. J. Pharmacol..

[17] Cui C.H., Kim D.J., Jung S.C., Kim S.C., Im W.T. (2017). Enhanced Production of Gypenoside LXXV Using a Novel Ginsenoside-Transforming β-Glucosidase from Ginseng-Cultivating Soil Bacteria and Its Anti-Cancer Property. Molecules.

[18] Wu F., Bian D., Xia Y., Gong Z., Tan Q., Chen J., Dai Y. (2012). Identification of Major Active Ingredients Responsible for Burn Wound Healing of Centella asiatica Herbs. Evid.-Based Complement. Altern. Med..

[19] Liu M., Dai Y., Li Y., Luo Y., Huang F., Gong Z., Meng Q. (2008). Madecassoside Isolated from *Centella asiatica* Herbs Facilitates Burn Wound Healing in Mice. Planta Med..

[20] Bindea G., Mlecnik B., Hackl H., Charoentong P., Tosolini M., Kirilovsky A., Fridman W.H., Pagès F., Trajanoski Z., Galon J. (2009). ClueGO: A Cytoscape plug-in to decipher functionally grouped gene ontology and pathway annotation networks. Bioinformatics.

[21] Dammeier J., Beer H.D., Brauchle M., Werner S. (1998). Dexamethasone is a novel potent inducer of connective tissue growth factor expression. Implications for glucocorticoid therapy. J. Biol. Chem..

[22] Attele A.S., Wu J.A., Yuan C.S. (1999). Ginseng pharmacology: Multiple constituents and multiple actions. Biochem. Pharmacol..

[23] Chen X. (1996). Cardiovascular protection by ginsenosides and their nitric oxide releasing action. Clin. Exp. Pharmacol. Physiol..

[24] Gillis C.N. (1997). Panax ginseng pharmacology: A nitric oxide link?. Biochem. Pharmacol..

[25] Nag S.A., Qin J., Wang W., Wang M.H., Wang H., Zhang R. (2012). Ginsenosides as anticancer agents: In vitro and in vivo activities, structure–activity relationships, and molecular mechanisms of action. Front. Pharmacol..

[26] Barrientos S., Stojadinovic O., Golinko M.S., Brem H., Tomic Canic M. (2008). Growth factors and cytokines in wound healing. Wound Repair Regen..

[27] Werner S., Grose R. (2003). Regulation of Wound Healing by Growth Factors and Cytokines. Physiol Rev..

[28] Pastore S., Mascia F., Mariani V., Girolomoni G. (2008). The Epidermal Growth Factor Receptor System in Skin Repair and Inflammation. J. Invest. Dermatol..

[29] Huang H., Cui W., Qiu W., Zhu M., Zhao R., Zeng D., Dong C., Wang X., Guo W., Xing W. (2015). Impaired wound healing results from the dysfunction of the Akt/mTOR pathway in diabetic rats. J. Dermatol. Sci..

[30] Carre A.L., Hu M.S., James A.W., Kawai K., Galvez M.G., Longaker M.T., Lorenz H.P. (2018). β-Catenin-Dependent Wnt Signaling: A Pathway in Acute Cutaneous Wounding. Plast. Reconstr. Surg..

[31] Huang C. (2004). MAP kinases and cell migration. J. Cell Sci..

[32] Houschyar K.S., Momeni A., Pyles M.N., Maan Z.N., Whittam A.J., Siemers F. (2015). Wnt signaling induces epithelial differentiation during cutaneous wound healing. Organogenesis.

[33] Squarize C.H., Castilho R.M., Bugge T.H., Gutkind J.S. (2010). Accelerated wound healing by mTOR activation in genetically defined mouse models. PLoS ONE.

[34] Greenhalgh D.G. (1996). The Role of Growth Factors in Wound Healing. J. Trauma Acute Care Surg..

[35] Borena B.M., Martens A., Broeckx S.Y., Meyer E., Chiers K., Duchateau L., Spaas J.H. (2015). Regenerative Skin Wound Healing in Mammals: State-of-the-Art on Growth Factor and Stem Cell Based Treatments. Cell. Physiol. Biochem..

[36] Sherbet G.V. (2011). Growth Factors and Their Receptors in Cell Differentiation, Cancer and Cancer Therapy.

[37] Henshaw F.R., Boughton P., Lo L., Mclennan S.V., Twigg S.M. (2015). Topically Applied Connective Tissue Growth Factor/CCN2 Improves Diabetic Preclinical Cutaneous Wound Healing: Potential Role for CTGF in Human Diabetic Foot Ulcer Healing. J. Diabetes Res..

[38] Leung K.W., Cheng Y.K., Mak N.K., Chan K.K.C., Fan T.P.D., Wong R.N.S. (2006). Signaling pathway of ginsenoside-Rg1 leading to nitric oxide production in endothelial cells. FEBS Lett..

[39] Leung K.W., Leung F.P., Huang Y., Mak N.K., Wong R.N.S. (2007). Non-genomic effects of ginsenoside-Re in endothelial cells via glucocorticoid receptor. FEBS Lett..

[40] Mohanan P., Subramaniyam S., Mathiyalagan R., Yang D.C. (2018). Molecular signaling of ginsenosides Rb1, Rg1, and Rg3 and their mode of actions. J. Ginseng Res..

[41] An D.S., Cui C.H., Siddiqi M.Z., Yu H.S., Jin F.X., Kim S.G., Im W.T. (2017). Gram-Scale Production of Ginsenoside F1 Using a Recombinant Bacterial β-Glucosidase. J. Microbiol. Biotechnol..

[42] Du J., Cui C.H., Park S.C., Kim J.K., Yu H.S., Jin F.-X., Sun C., Kim S.C., Im W.T. (2014). Identification and Characterization of a Ginsenoside-Transforming β-Glucosidase from Pseudonocardia sp. Gsoil 1536 and Its Application for Enhanced Production of Minor Ginsenoside Rg2(S). PLoS ONE.

[43] Siddiqi M.Z., Cui C.H., Park S.K., Han N.S., Kim S.C., Im W.T. (2017). Comparative analysis of the expression level of recombinant ginsenoside-transforming β-glucosidase in GRAS hosts and mass production of the ginsenoside Rh2-Mix. PLoS ONE.

[44] Cui C.H., Kim S.C., Im W.T. (2013). Characterization of the ginsenoside-transforming recombinant β-glucosidase from Actinosynnema mirum and bioconversion of major ginsenosides into minor ginsenosides. Appl. Microbiol. Biotechnol..

[45] Cui C.H., Liu Q.M., Kim J.K., Sung B.H., Kim S.G., Kim S.C., Im W.T. (2013). Identification and Characterization of a Mucilaginibacter sp. Strain QM49-Glucosidase and Its Use in the Production of the Pharmaceutically Active Minor Ginsenosides (*S*)-Rh 1 and (*S*)-Rg 2. Appl. Environ. Microbiol..

[46] Cui C.H., Kim J.K., Kim S.C., Im W.T. (2014). Characterization of a ginsenoside-transforming β-glucosidase from Paenibacillus mucilaginosus and its application for enhanced production of minor ginsenoside F2. PLoS ONE.

[47] Kim J.K., Cui C.H., Liu Q., Yoon M.H., Kim S.C., Im W.T. (2013). Mass production of the ginsenoside Rg3(S) through the combinative use of two glycoside hydrolases. Food Chem..

[48] Mahmood T., Yang P.C. (2012). Western blot: Technique, theory, and trouble shooting. N. Am. J. Med. Sci..

[49] Mccloy R.A., Rogers S., Caldon E., Lorca T., Castro A., Burgess A. (2014). Cell Cycle Partial inhibition of Cdk1 in G 2 phase overrides the SAC and decouples mitotic events. Cell Cycle.

