# Supplementary material for: Gypenoside LXXV Promotes Cutaneous Wound Healing In Vivo by Enhancing Connective Tissue Growth Factor Levels Via the Glucocorticoid Receptor Pathway
Source: Molecules. 2019 Apr 23;24(8):1595. doi: 10.3390/molecules24081595 (PMC6515290; doi:10.3390/molecules24081595)
Supplement: Supplementary file 1 [file molecules-24-01595-s001.pdf]

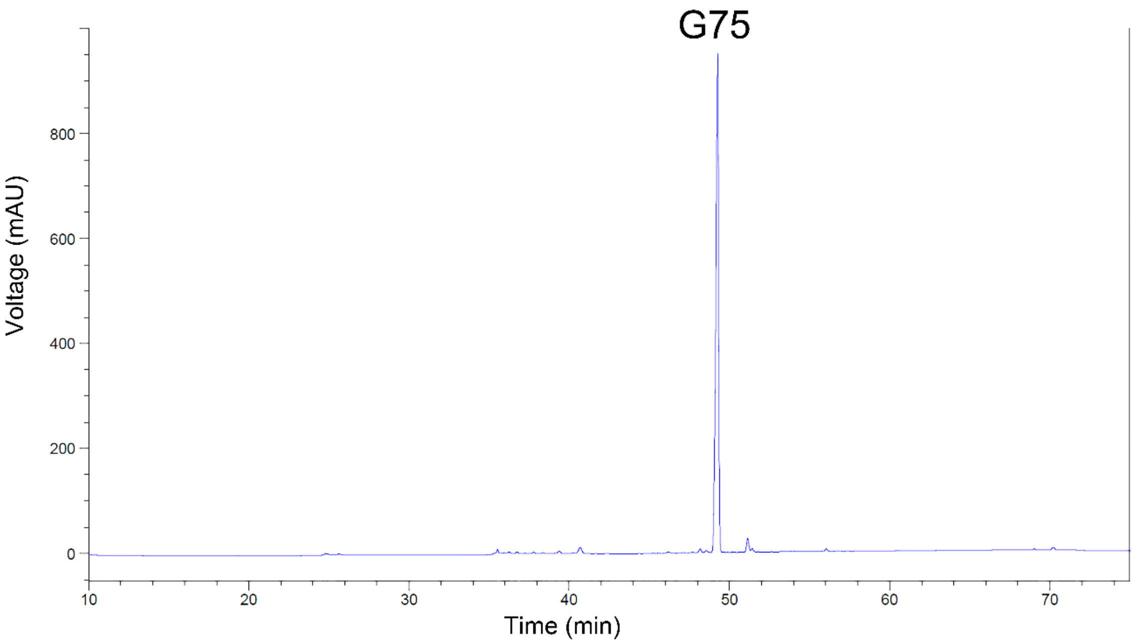

Supplementary Figure S1. HPLC results of the purified G75 (95.1%). The HPLC analysis was performed using an HPLC system (Younglin Co. Ltd, Korea). The separation was performed on a Prodigy ODS (2) C_{18} column (5 µm, 150 × 4.6 mm i.d., Phenomenex, CA, USA) with a guard column (Eclipse XDB C_{18}, 5 µm, 12.5 × 4.6 mm i.d., Agilent Technologies, CA, USA). The flow rate was 1.0 ml/min, and the detection was measured by monitoring the absorbance at 203 nm and with an injected volume of 25 µl.
